# Draft Genome Sequence of Aduncisulcus paluster, a Free-Living Microaerophilic Eukaryote Belonging to the Fornicata

**DOI:** 10.1128/mra.00539-22

**Published:** 2023-01-25

**Authors:** Ikuko Yuyama, Keitaro Kume, Takumi Tamura, Yuji Inagaki, Tetsuo Hashimoto

**Affiliations:** a Graduate School of Sciences and Technology for Innovation, Yamaguchi University, Yamaguchi, Japan; b Faculty of Medicine, University of Tsukuba, Ibaraki, Japan; c Graduate School of Life and Environmental Sciences, University of Tsukuba, Ibaraki, Japan; d Center for Computational Sciences, University of Tsukuba, Ibaraki, Japan; e Faculty of Life and Environmental Sciences, University of Tsukuba, Ibaraki, Japan; University of California, Riverside

## Abstract

Aduncisulcus paluster is a free-living, unicellular flagellate belonging to the eukaryotic lineage Fornicata, which includes free-living and commensal/parasitic organisms. Here, we report the draft genome sequence of *A. paluster*, which provides clues for elucidating the adaptation to microaerophilic/anaerobic environments and the transition between free-living and commensal/parasitic lifestyles in Fornicata.

## ANNOUNCEMENT

The Fornicata include various microaerophiles/anaerobes, which possess mitochondrion-related organelles. Aduncisulcus paluster (1) is a free-living fornicate that shares a common ancestor with the parasites (2). Previously, only transcriptome data were available for *A. paluster*, which contain many sequences derived from bacteria in the culture medium (2). Therefore, to obtain more accurate genetic information, we performed genome/transcriptome analyses on *A. paluster* cells purified by density gradient centrifugation (3). This is the third genomic analysis of free-living fornicates (3, 4).

We have maintained the laboratory culture of *A. paluster* NY0171, which is identical to the strain NIES-1843 (1). The culture containing its food bacteria was kept at 17.5°C in a modified TYGM-9 medium (10%) prepared with filtered seawater. We inoculated from a single *A. paluster* culture to fresh medium in three flasks (1,650 mL in total) and cultured the cells for 1 week. The cells in two out of the three flasks were combined and used for RNA extraction, and the cells in the other flask were applied to DNA extraction. The cells were collected by density gradient centrifugation using the 0, 10, and 20% Optiprep (Sigma) gradient at 17°C, 800 × *g*, for 20 min (3). The purified cells at 3.4 × 10^7^ and 1.56 × 10^6^ were used for RNA and DNA extractions, respectively. DNA was extracted using the SDS plus phenol-chloroform-isoamyl alcohol (25:24:1) method (5), while RNA was extracted using TRIzol reagent (Sigma) according to the manufacturer’s instructions. We constructed the libraries for transcriptome sequencing (RNA-seq) and genome sequencing (Genome-seq) analyses using the Kapa stranded mRNA-seq kit for RNA and the Kapa HyperPrep kit (Kapa Biosystem) for DNA, respectively. Prior to the library construction, the DNA sample was sheared into ≈500 bp by an ultrasonicator (Covaris). Then, Genome-seq and RNA-seq analyses (150-bp paired-end) were performed on HiSeqX and NextSeq500 (Illumina) at a biotech company (SeibutsuGiken Inc.).

We obtained 463,267,674 reads/69.9 Gbp (Genome-seq) and 223,709,682 reads/31.8 Gbp (RNA-seq). We used the default parameters for subsequent analyses unless otherwise specified. Reads were preprocessed using Fastp v0.21.0 with a >Q30 threshold (6), followed by *de novo* assembly performed in MaSuRCA v3.3.3 (Genome-seq) (7) and Trinity v2.12 (RNA-seq) (8). For gene prediction, we executed Braker2 v2.1.6 (9–15) with the supporting information generated by aligning the cleaned RNA-seq reads to the MaSuRCA assembly by TopHat v2.1.1 (16) (option: -g 1), and we used TransDecoder v5.5.0 (https://github.com/TransDecoder/TransDecoder) for the Trinity assembly. Based on the BLASTN/BLASTP (17, 18) searches using the assembled/predicted sequences against the NCBI nt/nr databases (ver. Dec-21-2021/Feb-04-2022), we removed the putative contaminated sequences that matched to the top hit database sequences from outside the phylum, Metamonada, that includes the Fornicata, with an E value of <1e−10 and pident (percentage of identical matches) of >0.95. Then, we assessed genome completeness of the predicted sequences by BUSCO v5.1.2 with the eukaryote ODB10 data set (19), and it was 40.4% (complete, 30.2%; fragment, 10.2%). We finally summarized the statistics in Table 1. Both predicted protein sequences were annotated by InterProScan v5.52 (20, 21) and by BLASTP search against nr (if pident was >0.9 and qcov (query coverage) was >0.9, the best-hit annotation was used).

**TABLE 1:** Overview of nuclear genome sequences for fornicates

Description	Genome size (Mb)	No. of contigs	*N*_50_ (kbp)	GC (%)	No. of predicted proteins	Mean amino acid (aa) length	No. of introns	Reference(s)
*Aduncisulcus paluster*	29.4	25,863	4.6	39.1	15,316	418.5	11,743	This study
Carpediemonas membranifera	24.2	69	905.8	57.1	8,300	467.2		4; this study
Kipferlia bialata	51.0	11,563	10.5	49.4	17,389	333.0	124,912	3
Spironucleus salmonicida	12.9	233	150.8	33.5	8,067	373.0	3	3, 4
Giardia intestinalis	12.8	211	2,762.4	49.2	5,901	530.0	8	3, 4

This draft genome will help in the study of the genome evolution associated with the evolutionary transition between free-living and commensal/parasitic lifestyles in the Fornicata.

### Data availability.

The genome/transcriptome sequences are available at DDBJ/ENA/GenBank (sra-run DRR351251/DRR353576) under accession no. BQXS01000001 to BQXS01023235/ICSK01000001 to ICSK01036959.
